# Defective structural RNA processing in relapsing-remitting multiple sclerosis

**DOI:** 10.1186/s13059-015-0629-x

**Published:** 2015-03-25

**Authors:** Charles F Spurlock, John T Tossberg, Yan Guo, Subramaniam Sriram, Philip S Crooke, Thomas M Aune

**Affiliations:** Department of Medicine, Vanderbilt University School of Medicine, Nashville, TN 37232 USA; Department of Cancer Biology, Vanderbilt University School of Medicine, Nashville, TN 37232 USA; Department of Neurology, Vanderbilt University School of Medicine, Nashville, TN 37232 USA; Department of Mathematics, Vanderbilt University, Nashville, TN 37232 USA; Department of Pathology, Microbiology and Immunology, Vanderbilt University School of Medicine, Nashville, TN 37232 USA; Medical Center North T3113, Vanderbilt University Medical Center, 1161 21st Avenue South, Nashville, TN USA

## Abstract

**Background:**

Surveillance of integrity of the basic elements of the cell including DNA, RNA, and proteins is a critical element of cellular physiology. Mechanisms of surveillance of DNA and protein integrity are well understood. Surveillance of structural RNAs making up the vast majority of RNA in a cell is less well understood. Here, we sought to explore integrity of processing of structural RNAs in relapsing remitting multiple sclerosis (RRMS) and other inflammatory diseases.

**Results:**

We employed mononuclear cells obtained from subjects with RRMS and cell lines. We used quantitative-PCR and whole genome RNA sequencing to define defects in structural RNA surveillance and siRNAs to deplete target proteins. We report profound defects in surveillance of structural RNAs in RRMS exemplified by elevated levels of poly(A) + Y1-RNA, poly(A) + 18S rRNA and 28S rRNAs, elevated levels of misprocessed 18S and 28S rRNAs and levels of the U-class of small nuclear RNAs. Multiple sclerosis is also associated with genome-wide defects in mRNA splicing. Ro60 and La proteins, which exist in ribonucleoprotein particles and play different roles in quality control of structural RNAs, are also deficient in RRMS. In cell lines, silencing of the genes encoding Ro60 and La proteins gives rise to these same defects in surveillance of structural RNAs.

**Conclusions:**

Our results establish that profound defects in structural RNA surveillance exist in RRMS and establish a causal link between Ro60 and La proteins and integrity of structural RNAs.

**Electronic supplementary material:**

The online version of this article (doi:10.1186/s13059-015-0629-x) contains supplementary material, which is available to authorized users.

## Background

Relapsing remitting multiple sclerosis (RRMS) affects approximately 0.1% of the population. RRMS is characterized by de-myelination of neurons but disease mechanisms are incompletely understood. Involvement of innate and adaptive arms of the immune system is detected in inflammatory demyelinating lesions implicating these in pathogenesis. Both genetic and environmental factors are also implicated in the origins of RRMS [1-8].

Studies have demonstrated an expanding complexity of non-protein-coding RNAs (ncRNA) in higher eukaryotes [9]. Non-vertebrate metazoans and microbes possess a single Y RNA while humans encode up to four Y RNAs, termed Y1, Y3, Y4, and Y5 RNA [10]. Y RNAs are approximately 100 nucleotides in length and are transcribed by RNA polymerase III. Y RNAs possess sequence-specific binding sites for the Ro60 protein [11]. All four human Y RNAs assemble into ribonucleoprotein (RNP) particles including Ro60, La, and other proteins [12]. Precise functions of Y RNAs and Ro nucleoprotein particles are incompletely understood but studies suggest they contribute to ncRNA quality control and ribosomal RNA (rRNA) processing [13,14]. To produce mature rRNAs, RNA polymerase 1 transcribes a 47S precursor rRNA that is processed via endonucleolytic, exonucleolytic, and additional modifications producing mature 5.8S, 18S, and 28S rRNAs [15-17]. Small nuclear U RNAs are another form of ncRNA making up the RNA portion of the RNP complex known as the spliceosome required for splicing of pre-mRNAs to mature mRNAs [18,19]. In vertebrates, major small nuclear U RNAs include U1, U2, U4, U5, and U6 [20,21]. Defects or mutations in specific small nuclear U RNAs or the genes encoding proteins required for biogenesis of U-RNA RNP complexes, such as SMN1 (survival of motor neuron 1), give rise to specific neurodegenerative disorders rather than global defects [22-26].

Polyadenylation of mRNAs at the 3’ end is a necessary step in their synthesis and maturation [14,19,27-32]. Mature, fully processed ncRNAs lack 3’ poly(A) tails. Addition of poly(A) tails to ncRNAs represents a critical quality-control step to promote degradation of mis-folded or mis-processed ncRNAs. Specific protein complexes exist to add poly(A) tails to RNA substrates to promote their degradation by stimulating exonucleolytic activity of the exosome thus leading to degradation of mis-processed or mis-folded ncRNAs.

It is fundamentally unknown how normal cellular processes or responses to extracellular stimuli may invoke polyadenylation and degradation of ncRNA substrates or if human disease processes exhibit defects in polyadenylation of ncRNA substrates as part of their pathogenesis. Here, our results demonstrate that mononuclear cells from subjects with RRMS exhibit pervasive increases in levels of polyadenylated ncRNAs including Y1 RNA, 18S and 28S rRNA, and U1, U2, and U4 snRNAs and these defects are unique to RRMS. Defects in expression of both Ro60 and La proteins in RRMS appear to contribute to increased polyadenylation of ncRNAs. Further, IFN-β1b, a common RRMS therapy [33], restores both Ro60 and La levels to normal as well as levels of polyadenylated Y1 RNA and U1 snRNA suggesting that aberrant polyadenylation of ncRNA substrates may have pathogenic consequences.

## Results

### Elevated polyadenylation of ncRNA substrates in RRMS

**Figure 1:**
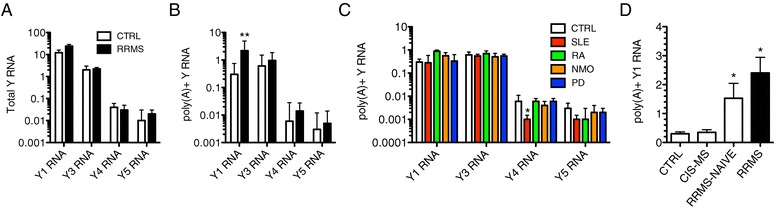
Increased levels of poly(A) + Y1 RNA in RRMS. (**A**) Total Y RNA levels in peripheral whole blood determined after cDNA synthesis using random hexamers in CTRL (N = 24) and RRMS (N = 22). (**B**) As in (A) except oligo-dT was used for cDNA synthesis. (**C**) As in (B) except different disease cohorts. Individual transcripts were normalized to *GAPDH* transcript levels in CTRL (N = 24), SLE (N = 24), RA (N = 18), NMO (N = 22), and PD (N = 19). (**D**) Total poly(A) + Y1 RNA levels were determined using oligo-dT in CTRL (N = 24), CIS-MS (N = 16), RRMS-NAÏVE (N = 24), and RRMS (N = 22). Error bars are S.D. *P <0.05, **P <0.005.

To initiate our studies, we determined levels of Y RNAs in different subject cohorts by quantitative PCR using either random hexamers or oligo-dT for synthesis reasoning that random hexamers should allow amplification of all Y RNAs independent of whether or not they were polyadenylated but that oligo-dT would allow amplification of only those Y RNAs that were polyadenylated consistent with previously established methodologies [31]. We also confirmed that >95% of total cellular Y RNAs were retained during the PaxGene total RNA isolation procedure. Sanger sequencing of the PCR products confirmed identity of all human Y RNAs. We found that levels of total Y RNAs (random hexamers) were not markedly different between CTRL and RRMS subjects (Figure 1A). In marked contrast, levels of polyadenylated Y1 RNA (oligo-dT), but not other Y RNAs, were increased by about 20-fold in RRMS relative to CTRL (Figure 1B). This elevation of polyadenylated Y1 RNA was not seen in systemic lupus erythematosus (SLE), rheumatoid arthritis (RA), neuromyelitis optica (NMO), or Parkinson’s disease (PD) (Additional file 1: Table S1 for a description of subjects) (Figure 1C). In a cross-sectional analysis, we compared levels of polyadenylated Y1 RNA in blood samples obtained from subjects experiencing a first clinically isolated syndrome who went on to develop RRMS at a later date (CIS-MS), from subjects at the time of their diagnosis of RRMS but prior to onset of any therapies (RRMS-NAÏVE) and from subjects with RRMS of >1 year’s duration (RRMS) (Figure 1D). We found that levels of polyadenylated Y1 RNA were elevated in both RRMS-NAÏVE and RRMS cohorts but that levels of polyadenylated Y1 RNA were similar to CTRL levels in the CIS-MS cohort. Thus, increased polyadenylation of Y1 RNA, but not other Y RNAs, was observed in RRMS, but not other autoimmune diseases.

**Figure 2:**
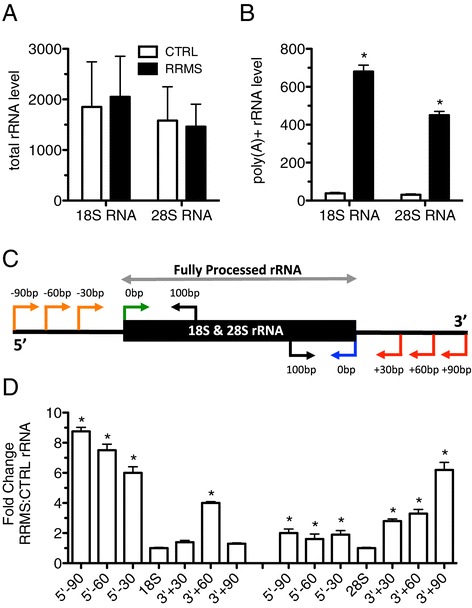
Increased polyadenylation and mis-processing of 18S and 28S rRNAs in RRMS. (**A**) Total rRNA levels in blood determined after cDNA synthesis using random hexamers in CTRL (N = 24) and RRMS (N = 22). (**B**) As in (A) except oligo-dT was used for cDNA synthesis. (**C**) Schematic illustrating PCR strategy to detect misprocessed 18S and 28S rRNAs. (**D**) Transcript levels of misprocessed rRNAs (-90, -60, -30) in RRMS (N = 12) relative to CTRL (N = 12). Results were normalized to total 18S or 28S RNA and are presented as fold difference between CTRL and RRMS. *P <0.05.

We used a similar approach to measure levels of total and polyadenylated 18S and 28S rRNAs. We found that levels of total 18S and 28S rRNAs were similar between the RRMS and CTRL cohorts when random hexamers were used for cDNA synthesis (Figure 2A). However, we found a marked increase in levels of both polyadenylated 18S and 28S rRNAs as measured by employing oligo-dT for cDNA synthesis (Figure 2B). We therefore determined if there was also an increase in misprocessed rRNAs in RRMS since rRNAs are polyadenylated in the presence of defective processing of the 18S and 28S rRNAs from their 47S rRNA precursor. We predicted that misprocessing of rRNAs should produce 18S and 28S rRNAs longer than the typical 1.9 kb and 5.0 kb, respectively. To test this hypothesis, we designed a series of PCR primers to probe lengths of 18S and 28S rRNAs. We used common primers within the 18S or 28S rRNA (black arrows) and four additional primers; one within the classic 18S or 28S rRNA (green and blue arrows) and three that extend 30, 60, or 90 bp beyond the length of the fully processed 1.9 kb 18S or 5 kb 28S rRNA transcript (orange and red arrows) (Figure 2C). Random hexamers were used for cDNA synthesis. We found that 18S and 28S transcript levels in CTRL and RRMS were approximately the same (normalized to 1), labeled 18S (Figure 2D, left) or 28S (Figure 2D, right), when PCR primers within the 1.9 kb 18S rRNA or 5.0 kb 28S rRNA were employed. However, when we employed an internal primer (black arrow) and external primers 30, 60, or 90 bp distal to the normal transcript size (orange arrows, red arrows) we found a marked increase in transcript levels of 18S rRNA with extended ends between 30 and 90 bp in RRMS compared to CTRL. These 18S rRNA extended ends were more frequent at the 5’ end of the 18S rRNA than the 3’ end of the 18S rRNA. The 28S rRNA had only a modest increase in extended ends in RRMS relative to CTRL at the 5’ end. For the 28S RNA species, the increase in the frequency of extended ends in RRMS relative to CTRL was more pronounced at the 3’ end. We interpret these results to demonstrate that both 18S rRNAs and 28S rRNAs were not accurately processed to their mature size in RRMS.

**Figure 3:**
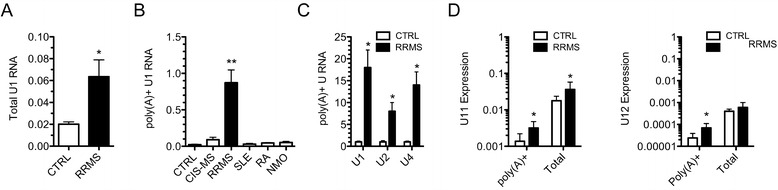
Increased total and polyadenylated U snRNAs in RRMS. (**A**) Total U1 RNA transcript levels were determined by quantitative PCR using random hexamers for cDNA synthesis in CTRL (N = 24) and RRMS (N = 22). Individual transcripts were normalized to *GAPDH* transcript levels. (**B**) As in (A) except total poly(A) + U1 snRNA levels were determined by quantitative PCR using oligo-dT for cDNA synthesis in CTRL (N = 24), CIS-MS (N = 16), RRMS (N = 22), SLE (N = 24), RA (N = 18), and NMO (N = 22). (**C**) As in (A), except transcript levels were determined by whole genome RNA-sequencing (RNA-seq) and normalized to CTRL = 1; CTRL (N = 8) and RRMS (N = 6). (**D**) As in (A, B) except transcript levels of U11 and U12 snRNAs were determined by quantitative PCR using oligo d(T) for poly(A) + or random hexamer primers for total U11 or U12 snRNA.

Next, we determined levels of U1 RNA in blood (PaxGene tubes) from CTRL subjects, subjects with RRMS or with other inflammatory diseases. As above, synthesis of cDNA was performed using random hexamers and oligo-dT and transcript levels were determined by quantitative PCR. We found a modest increase in total levels of U1 RNA (random hexamers) in RRMS relative to CTRL (Figure 3A). In contrast, poly(A) + U1 transcript levels were markedly elevated in subjects with RRMS relative to CTRL (Figure 3B). The CIS-MS cohort did not exhibit increased poly(A) + U1 RNA levels nor did subjects with other inflammatory diseases, SLE, RA, and NMO. We also extracted RNA-seq data and confirmed that poly(A) + U1 RNA levels were elevated in RRMS and that U2 and U4 RNA levels were also elevated in RRMS relative to CTRL (Figure 3C). We also examined expression of total and poly(A) + U11 and U12 transcripts, components of the minor spliceosome pathway, and found elevated poly(A) + U11 and U12 transcript levels in RRMS versus CTRL and increased total U11 transcript levels. Total levels of U12 snRNA were not significantly different between RRMS and CTRL (Figure 3D). Thus, multiple ncRNA species exhibit increased polyadenylation in RRMS relative to CTRL or other autoimmune diseases.

### Alterations in mRNA processing in RRMS

**Figure 4:**
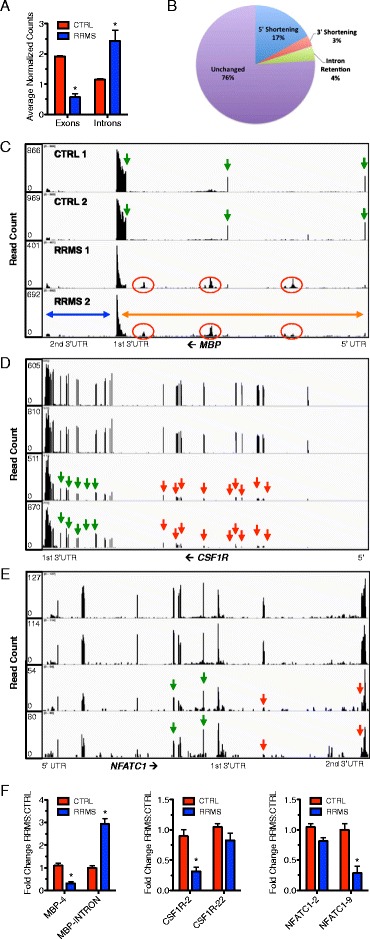
Extensive mRNA isoform loss and intron retention in RRMS. (**A**) Total exon loss and intron gain events across the genome in RRMS (N = 6) and CTRL (N = 8) were determined from RNA-seq analysis (see Methods). Results are expressed as average normalized counts or reads across known exons and introns in CTRL and RRMS. (**B**) Percent abundance of transcript alterations in RRMS relative to CTRL PBMC determined from analysis of genome-wide RNA-seq data. (**C**) Example of intron retention in mRNA encoded by *MBP*. Green arrows identify exons. Red circles identify retention of intron sequences in mature mRNA in RRMS. Orange and blue arrows identify two isoforms. (**D**, **E**) Examples of 5’ mRNA shortening, *CSF1R*, and 3’ mRNA shortening, *NFATC1* in RRMS. Red arrows identify loss of exons in RRMS and green arrows identify exons expressed at equal levels between CTRL and RRMS. (**F**) Expression levels of individual exons and the MBP intron in CTRL (N = 12) and RRMS (N = 12) was determined by quantitative PCR and normalized to CTRL = 1.0 after normalization to transcript levels of *GAPDH*, error bars are S.D. * = *P* <0.05.

The U1, U2, U4, U5, and U6 RNAs play critical roles in determining mRNA length and isoform expression via several mechanisms including protection from premature cleavage and polyadenylation of nascent pre-mRNAs [21,26,34]. Disruption of levels of individual U RNAs is sufficient to cause global alterations in alternative splicing and mRNA length. It is not known how imbalances or altered polyadenylation of multiple U snRNAs may impact alternative splicing and mRNA lengths. Since these defects include mRNA shortening by loss of 5’ or 3’ exons, choice of alternative 5’ and 3’ UTRs, choice of initial and final exons and partial retention of intron sequences, we searched our RNA-seq data for differences in splicing and intron retention between CTRL subjects and subjects with RRMS. For each chromosome, we counted the number of reads per exon across the genome in CTRL and RRMS subjects. We found a genome-wide loss of exon expression in RRMS relative to CTRL (Figure 4A). Genome-wide analysis of expressed genes demonstrated that 17% of transcribed genes exhibited loss of 5’ exons (5’ shortening) (Figure 4B). Loss of 3’ exons (3’ shortening, 3% of transcribed genes) was less frequent. Intron retention was also less frequent and only 4% of transcribed genes exhibited forms of intron retention. Visual inspection of 3,000 or 30% of expressed genes using the Integrative Genome Viewer (IGV) confirmed these results. We defined 5’ and 3’ shortening to mean that at least one exon at the 5’ or 3’ end of the transcript, respectively, exhibited a >5-fold reduction in expressed counts or FPKM relative to exons at the 3’ or 5’ end of the transcript, respectively, in RRMS subjects compared to CTRL subjects. Intron retention was similarly defined as an increase in read counts localized to an intron by >5-fold in RRMS compared to CTRL subjects. The majority of genes transcribed in mononuclear cells from RRMS compared to CTRL did not display differences in isoform distribution or retention of intron sequences.

We analyzed the number of read counts per intron and found that subjects with RRMS exhibited higher intron read counts across chromosomes compared to CTRL. Examples of these alterations included *MBP*, *CSF1R*, and *NFATC1* (Figure 4C to E). We found that the myelin basic protein gene, MBP, which encodes both classic myelin basic protein expressed primarily by myelin forming cells and a second family of proteins, called golli proteins, expressed by T lymphocytes, neurons, and oligodendrocytes, displayed increased intron retention in the mature mRNA (Figure 4C, note that transcription is from right to left) [35-37]. Individual exons are labeled with green arrows and regions of intron retention seen in RRMS are labeled with red circles. Retained intron sequences were present in both ‘golli’ and ‘golli-MBP’ mRNAs; classic MBP gene depicted by the blue arrow, golli-MBP gene depicted by the orange arrow. Thus, U snRNA imbalance in RRMS was associated with intron retention in MBP mRNA.

We also found a marked reduction in transcript levels of the exons comprising *CSF1R* mRNAs. In CTRL subjects, each of the 22 exons was present at approximately equal abundance. However, in RRMS, there was decreased transcript abundance of exons 1 to 11 (green arrows identify exons expressed at similar levels in CTRL and RRMS and red arrows show exon loss) (Figure 4D). Thus, U snRNA imbalance in RRMS was also associated with 5’ shortening. 3’ shortening was also found in RRMS (Figure 4E). All nine exons of NFATC1 mRNA and the 3’ UTR exhibited similar transcript abundance assembled into a continuous mRNA in CTRL subjects. RRMS subjects exhibited a specific loss of the eighth and ninth exons (note that the ninth exon is continuous with the 3’ UTR, red arrows). The remaining expressed exons were present at similar levels in CTRL and RRMS (green arrows). We then validated these findings in a different cohort of CTRL and RRMS subjects using quantitative PCR (Figure 4F). Transcript levels of MBP exon 4, CSF1R exon 2, and NFATC1 exon 9 were significantly reduced in RRMS versus CTRL. Levels of CSF1R exon 22 and NFATC1 exon 2 were only modestly reduced in RRMS similar to our RNA-sequencing findings. We also found increased transcript levels of the MBP intron between MBP exons 3 and 4 in RRMS compared to CTRL. Thus, using both quantitative PCR and RNA-sequencing, we were able to confirm specific examples of intron retention, 5’ shortening, and 3’ shortening in this independent sample set of RRMS subjects. These shortened mRNA isoforms seen in RRMS are consistent with mRNA premature cleavage and polyadenylation, a property that is produced by imbalances of U snRNAs or loss of U snRNA function. U snRNA imbalance observed in RRMS may contribute to intron retention as well as exon loss and mRNA shortening.

### Reduced expression of Ro60 and La in RRMS

**Figure 5:**
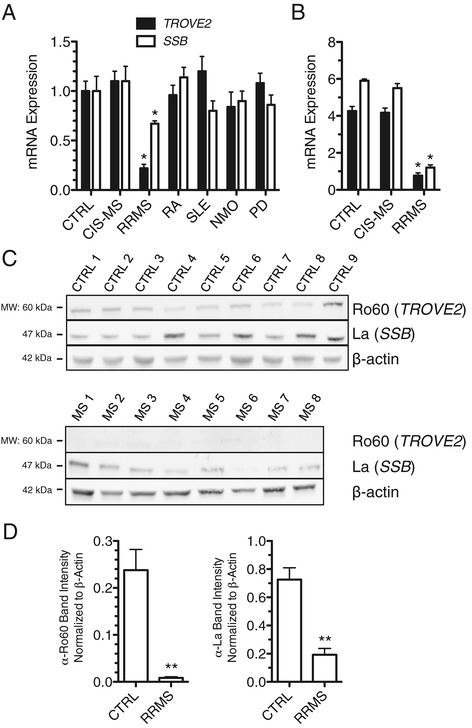
Ro60 and La proteins are depressed in RRMS. (**A**) Ro60 (*TROVE2*) and La (*SSB*) transcript levels in CTRL (N = 24), CIS-MS (N = 16), RRMS (N = 22), RA (N = 18), SLE (N = 24), NMO (N = 22), and PD (N = 19) were determined by quantitative PCR after cDNA synthesis using oligo-dT. Results are normalized to CTRL = 1.0 after normalization to transcript levels of *GAPDH*, error bars are S.D. (**B**) As in (A) using whole genome RNA-sequencing data. (**C**) Western blotting to determine Ro60 and La protein levels in PBMC from CTRL (N = 9) and RRMS (N = 8). (**D**) Quantitative estimates of protein abundance relative to β-actin. *P <0.05, **P <0.01.

Ro60 and La proteins are components of ribonucleoprotein particles, bind discrete structural ncRNAs, and are thought to play important roles in ncRNA processing and quality control. For these reasons, we measured *TROVE2* (Ro60) and *SSB* (La) expression levels in blood samples harvested in PaxGene tubes from the following cohorts of subjects: CTRL, CIS-MS, RRMS, RA, SLE, NMO, and PD. We found that *TROVE2* and *SSB* transcript levels were markedly reduced in the established RRMS cohort compared to CTRL. This difference was unique to RRMS and not observed in other autoimmune disease cohorts or in other inflammatory (NMO) or non-inflammatory (PD) neurologic conditions (Figure 5A). We replicated these findings by whole-genome RNA sequencing (RNA-seq) and obtained equivalent results (Figure 5B). We also determined levels of protein expression of Ro60 and La in PBMC by western blotting. We found that both Ro60 and La proteins were profoundly diminished in RRMS PBMC relative to CTRL PBMC (Figure 5C and D). Thus, both *TROVE2* and *SSB* transcripts and Ro60 and La proteins were profoundly diminished in RRMS and these mRNA and protein expression differences were not seen in several other autoimmune diseases.

### Interferon-β1b (Betaseron) and structural defects in RRMS

**Figure 6:**
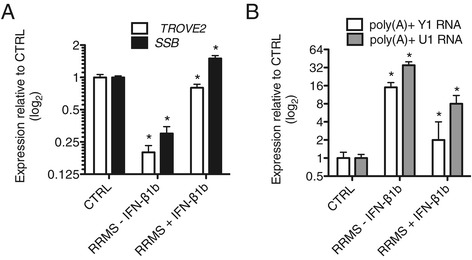
Interferon-β1b (IFN-β1b) therapy corrects aberrant levels of *TROVE2*, *SSB*, poly (A) + Y1 RNA, and poly(A) + U1 snRNA in RRMS. Blood samples were collected in PaxGene tubes from CTRL (N = 12), RRMS subjects not on IFN-β1b (RRMS - IFN-β1b, N = 12), and RRMS subjects on stable IFN-β1b therapy (RRMS + IFN-β1b, N = 4). Oligo dT was used for cDNA synthesis. Transcript levels of *TROVE2* and *SSB* (**A**) or poly(A) + Y1 RNA and poly(A) + U1 RNA (**B**) were determined by quantitative PCR and normalized to CTRL = 1 after normalization to levels of *GAPDH*. Error bars are ± S.D. *P <0.05.

For all analyses above, RRMS subjects were either on copaxone or no immunomodulatory therapy. Thus, we compared these subjects to RRMS on stable betaseron therapy. We found that levels of *TROVE2*, *SSB*, poly(A) + Y1 RNA and poly(A) + U1 snRNA were close to CTRL levels in RRMS subjects on betaseron therapy compared to RRMS subjects on either copaxone or no immunomodulatory therapy (Figure 6A, B). We examined responses of three individuals in longitudinal studies who initiated betaseron and found that correction of levels of *TROVE2*, *SSB,* poly(A) + Y1 RNA and poly(A) + U1 RNA was very rapid. We hypothesize that signaling pathways either directly or indirectly activated by betaseron interfere with signaling pathways driving defects in polyadenylation of structural RNAs in and expression of *TROVE2* and *SSB* in RRMS and that betaseron will be a useful tool to identify underlying mechanisms. Further, measurement of polyadenylated species of ncRNAs may provide a useful means to monitor responses to betaseron or other immunomodulatory therapies in RRMS.

### TROVE2 and SSB silencing disrupts structural RNA surveillance and alters mRNA length

**Figure 7:**
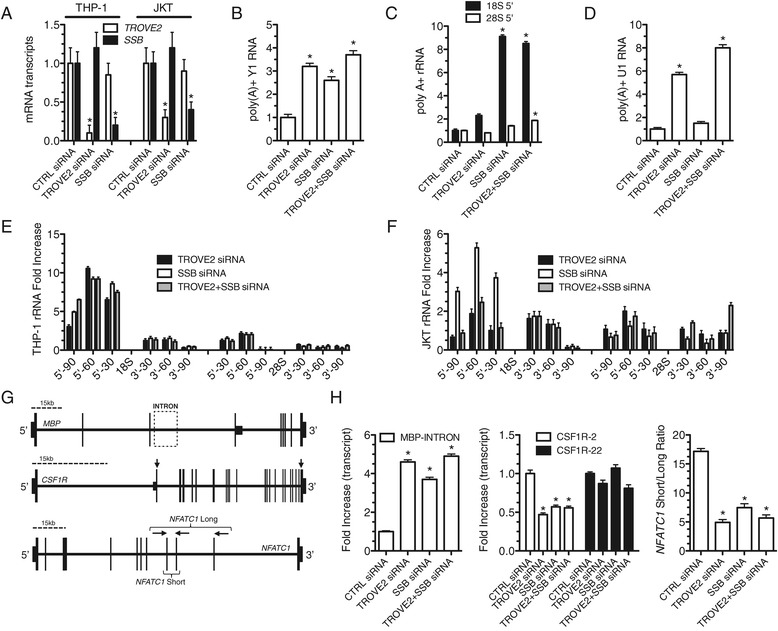
Loss of *TROVE2* and/or *SSB* via small RNA interference increases poly(A) + Y1 RNA, rRNAs, and U1 RNA, misprocessed 18S and 28S rRNA, and alters mRNA length. (**A**) Selective siRNA-mediated knockdown of *TROVE2* or *SSB* in THP-1 cells or Jurkat T cells. Transcript levels of TROVE2 and SSB were determined by quantitative PCR using oligo d(T) for cDNA synthesis. Results are normalized to cells transfected with a non-specific scrambled siRNA negative control after normalization to transcript levels of *GAPDH*. (**B**) Poly(A) + Y1 RNA levels were determined by quantitative PCR using oligo-dT for cDNA synthesis. Results normalized to CTRL (transfection of scrambled siRNA). (**C**) As in (*B*) except levels of 18S and 28S rRNAs were determined. (**D**) As in (B) except levels of poly(A) + U1 snRNA were determined. (**E**, **F**) Transcript levels of misprocessed rRNAs in THP-1 (E) and Jurkat T cells (F) (-90, -60, -30 bp) were determined and are expressed relative to cells transfected with a scrambled siRNA as described in Figure 2. (**G**) PCR strategy to test altered lengths of *MBP*, *CSF1R*, and *NFATC1* transcripts. (**H**) As in (A) except transcript levels of the MBP intron, CSF1R exons, and NFATC1 short to long mRNA isoform ratios were determined by quantitative PCR and normalized to CTRL. Each experiment was performed a minimum of three times, results are expressed as mean ± S.D *P <0.05.

To establish causal links between Ro60 and La imbalance and polyadenylation and processing of structural ncRNAs observed in RRMS, we designed siRNAs specific for human *TROVE2* and *SSB* and transfected them into the human THP-1 monocyte or the Jurkat T cell line. Transfection with *TROVE2* siRNA caused specific reduction of *TROVE2* transcripts but not *SSB* transcripts (Figure 7A). Similarly, the *SSB* siRNA caused specific loss of *SSB* transcripts but not *TROVE2* transcripts. We asked if reduced *TROVE2* or *SSB* levels resulted in an increase in the amount of poly(A) + Y1 RNA in THP-1 cells. We found that knockdown of either *TROVE2*, *SSB*, or the combination effectively increased levels of poly(A) + Y1 RNA (Figure 7B). Additionally, knockdown of *SSB* resulted in marked accumulation of poly(A) + 18S rRNA (Figure 7C). Knockdown of *TROVE2* resulted in only a modest increase in poly(A) + 18S RNA. In contrast, levels of poly(A) + 28S rRNA were largely unaffected by knockdown of either *TROVE2* or *SSB* but were modestly increased by the knockdown of both *TROVE2* and *SSB*. We also examined the impact of *TROVE2* or *SSB* knockdown on levels of poly(A) + U1 RNA and found that knockdown of *TROVE2*, but not knockdown of *SSB*, caused a marked increase in levels of poly(A) + U1 RNA (Figure 7D). rRNA misprocessing was also analyzed as described above. In both THP-1 cells and Jurkat cells, transfection of *TROVE2* and/or *SBB* siRNAs increased levels of misprocessed 18S rRNA and, to a lesser extent, misprocessed 28S rRNA (Figure 7E, F). Since the combination of *TROVE2* and *SSB* knockdown increased the amount of poly(A) + U1 RNA and alterations in levels of U1 RNA result in isoform switching, we determined if *TROVE2* and *SSB* RNA knockdown was sufficient to alter mRNA lengths in a cell [21]. We transfected *TROVE2* and *SSB* siRNAs into THP-1 cells and employed a PCR strategy to test for different lengths of *MBP*, *CSF1R*, and *NFATC1* transcripts (Figure 7G). Similar to what we observed *in vivo*, we found that reduced levels of either Ro60 or La by siRNA silencing were sufficient to increase expression levels of the *MBP* intron, to reduce expression of CSF1R exon 2 but not CSF1R exon 22, and also decrease the ratio of short to long *NFATC1* isoforms (Figure 7H). Finally, we examined the ability of Ro60 and La to bind these target mRNAs using RNA immunoprecipitation and quantitative PCR. Only a modest fraction of total CSF1R, NFATC1, or MBP mRNA bound to Ro60 or La (Additional file 2: Figure S1). Taken together, these results demonstrate that Ro60 and La protein levels are critical for many aspects of proper RNA surveillance including maintaining low levels of the poly(A) + ncRNAs, Y1 RNA, rRNAs, and U RNA, rRNA processing, and proper mRNA splicing; key components of RNA function in the cell.

## Discussion

We have found that extensive polyadenylation of structural RNAs, including Y1 RNA, U RNAs, and rRNAs is a feature of mononuclear cells from subjects with RRMS but not from subjects with other autoimmune or neurodegenerative conditions. Increased polyadenylation of rRNAs is associated with mis-processing of both 18S and 28S rRNAs. Alterations in polyadenylation and total U RNAs, essential components of the spliceosome, are associated with extensive 5’ shortening, 3’ shortening, and intron retention of mRNAs. Ro60 and La, two protein components of ribonucleoprotein complexes in the cell, are under-expressed in RRMS and siRNA-mediated ‘knockdown’ of Ro60 and/or La recapitulates these effects in cell lines resulting in increased polyadenylation of structural RNAs, misprocessing of 18S and 28S rRNAs, and isoform switching of mRNAs. Finally, IFN-β1b or betaseron, a common therapy for RRMS, but not other autoimmune diseases, restores the balance of polyadenylated structural RNAs to normal and reverses the loss of Ro60 and La that is seen in RRMS.

A general model is that misfolded or misprocessed RNA substrates are polyadenylated in ribonucleoprotein particles, termed the *Tr*f4p/*A*ir2p/*M*tr4p polyadenylation complex or TRAMP complex in yeast [29]. Trf4p and Trf5p are poly(A) polymerases. Mammalian orthologues include PAPD5 and PAPD7. This complex or similar complexes in higher eukaryotes catalyzes oligoadenylation or polyadenylation of RNA substrates, which, in turn, stimulates the exosome to degrade these poly(A) + substrates. Thus, in RRMS mononuclear cells, alterations in levels of misfolded or misprocessed structural RNAs or increased activity of the TRAMP complex or its equivalent may lead to increased polyadenylation of structural RNA substrates. Alternatively, decreased function of the exosome may lead to increased levels of poly(A) + structural RNA substrates. In fact, the eubacterium orthologue of Ro60 functions with Y RNA and the exosome to degrade misprocessed or misfolded structural RNAs [38]. Thus, defects in expression of Ro60 in RRMS may lead to defective function of the exosome and contribute to increased levels of polyadenylated structural RNAs seen in RRMS. Future studies will be required to delineate among these possibilities.

Ribosomal RNA processing proceeds via both endonucleolytic cleavages and exonucleolytic processing to yield mature 18S and 28S rRNAs. Levels of misprocessed rRNAs in cells are extremely low if effective RNA surveillance mechanisms outlined above are fully functional. Various manipulations, such as inhibition of RNA polymerase 1 by low concentrations of actinomycin D [30], produce aberrant pre-rRNA transcripts that are polyadenylated. Thus, abortive synthesis and processing of 18S and 28S rRNAs as well as other structural RNAs may also contribute to increased levels of polyadenylated structural RNAs in RRMS.

The U RNAs are another class of structural RNAs. These snRNAs constitute the RNA portion of the spliceosome required to remove introns from pre-mRNAs to produce mature mRNAs for translation. Polyadenylated forms of U1, U2, and U4 RNAs, as well as U11 and U12 components of the minor spliceosome pathway, are markedly increased in RRMS. There is also genome-wide disruption of splicing and processing of mRNAs resulting in 5’ and 3’ mRNA shortening and intron retention. An example of intron retention is the *MBP* gene. MBP has long been considered a candidate autoantigen for RRMS. In animal models, injection of foreign MBP gives rise to cross-reactivity to native MBP and inflammation, autoimmunity and clinical symptoms similar to human RRMS [39]. In a similar vein, intron retention in the MBP mRNA could produce MBP proteins with altered C-termini viewed by the immune system as foreign. Resulting immune responses to these altered MBP proteins could lead to cross-reactivity with native MBP and immune attack against myelin in the central nervous system thus contributing to development of RRMS.

Ro60 and La proteins are components of ribonucleoprotein particles. Both proteins bind certain structural RNAs. For example, Ro60 binds Y RNAs while La binds tRNAs, certain rRNAs, and microRNAs [13]. Both proteins are thought to play important roles in surveillance and quality control of structural RNAs. We found that both Ro and La proteins exhibit substantially reduced expression in RRMS but not other autoimmune diseases or other neurodegenerative diseases. In cell lines, we found that siRNA-mediated depletion of either Ro60 or La or both recapitulated many of the defects in structural RNA surveillance seen in RRMS mononuclear cells. Thus, depletion of Ro60 and La proteins in RRMS probably contributes to loss of structural RNA quality control in RRMS. Exact mechanisms are not completely clear. Ribonucleoprotein particles are heterogeneous complex structures that contain multiple proteins and RNA species with different functions. Loss of Ro60 and/or La may disrupt these ribonucleoprotein particles leading to loss of function, some of which are intimately involved in structural RNA quality control. Loss of Ro60 or La may also produce other defects in polyadenylation and/or degradation of structural RNAs. Further studies will be required to better understand mechanistic relationships between Ro60 and/or La deficiency and polyadenylation and processing of structural RNAs.

The relationship between deficiencies of Ro60 and La proteins in RRMS, defective quality control of structural RNAs in RRMS and disease pathogenesis is not immediately apparent. It is noteworthy that deletion of the Ro60 orthologue in mice results in selective development of a lupus-like autoimmunity syndrome including increased photosensitivity, membranoproliferative glomerulonephritis and production of autoantibodies suggesting that one role of Ro60 may be to prevent certain forms of autoimmunity [40]. Loss of Ro60 orthologues in various species also results in aberrant responses to UV irradiation, a form of cellular stress, defective ribosome biogenesis, including rRNA biogenesis, results in activation of stress responses mediated via the p53 pathway [41]. Numerous studies have implicated aberrant stress responses in the genesis of autoimmunity. This interpretation is consistent with our results that show that Ro60 and La deficiency are associated with human RRMS. It is also noteworthy that therapy IFN-β1b (betaseron), a common treatment for RRMS, restores levels of Ro60 and La proteins and polyadenylated structural RNA substrates to near normal. As such, monitoring levels of Ro60 and La proteins and polyadenylated structural RNA substrates in response to therapy may be useful predictors of disease activity or progression. It may also be informative to determine how levels of Ro60 and La proteins and polyadenylated structural RNA substrates respond to newer therapies for RRMS, such as Tysabri™, that are designed to keep mononuclear cells out of the central nervous system [42]. Studies such as these may define whether stimuli driving loss of Ro60 and La and accumulation of polyadenylated structural RNAs are derived from the central nervous system or the periphery.

It may seem unexpected that defects in expression of Ro60 and La proteins and defective structural RNA processing are seen in RRMS but not other autoimmune diseases. In some respects this is reminiscent of the findings that selective defects in RNA metabolism that are thought to be causative for specific neurodegenerative diseases [43-45]. In large part, these neurodegenerative diseases result from death of specific classes of neurons but are often caused by mutations in ubiquitously expressed genes but produce a very specific neurologic deficit. For example, spinal muscular atrophy is caused by deletions or mutations in the gene that encodes survival of motor neuron 1 (*SMN1*) and the SMN1 protein is ubiquitously expressed and plays a critical role in assembly of the spliceosome and mRNA processing. Similarly, defects in spliceosome integrity are also associated with the motor neuron disease amyotrophic lateral sclerosis. Other examples exist where defects in these universal cellular processes give rise to unique neurodegenerative disorders. It is also relevant to note that we have not examined defects in mRNA processing in other inflammatory or autoimmune disorders and these studies are in progress. It cannot be ruled out that integrity of mRNA processing is a common defect in multiple autoimmune diseases that may arise from different molecular pathways.

## Conclusion

Origins of defects in expression of Ro60 and La proteins, structural RNA processing, and mRNA processing in RRMS may arise from inheritance or from environmental events or responses to environmental events. Our cross-sectional analyses demonstrate that these defects are not seen in subjects at the time of their initial CIS event who go on to develop RRMS but are seen in subjects at their initial diagnosis of RRMS. The period of time between an initial CIS event and diagnosis of RRMS is quite variable but can be up to five years (1 to 4). For these reasons, we do not believe that defects described here arise via inheritance but rather propose that environmental events or responses to environmental changes are causative. We cannot rule out the possibility that these defects arise via somatic mutation and this will be the subject of future investigations. Further, if other cells, especially cells within affected tissues in the CNS, exhibit these defects is unknown and will be the subject of future investigations.

Finally, biologic pathways that control surveillance and quality control of structural RNAs are largely conserved throughout evolution. Our results show that acquired defects in these pathways are associated with human disease and may pave the way for a deeper understanding of how these pathways may be involved in normal biologic processes such as responses to extracellular and intracellular stimuli, responses to various forms of stress, and contributions to human disease.

## Materials and methods

### Study populations

Our patient cohort contained control subjects with no major medical conditions, chronic or acute infections, and no family history of autoimmune disease. Blood samples in PaxGene tubes were obtained from (1) patients with clinically isolated syndrome prior to diagnosis of RRMS; (2) at the time of diagnosis of RRMS before the onset of therapy; and (3) established RRMS of >1 year’s duration on medications. We also analyzed subjects (4) meeting the American College of Rheumatology (ACR) clinical criteria of RA; (5) meeting the ACR criteria for SLE; (6) NMO; and (7) PD. RRMS and NMO samples were obtained from nine sites (AZ, CA, MD, MA, MS, NY, SC, TN, TX)] in the United States with the assistance of the Accelerated Cure Project. In the established multiple sclerosis cohort, seven patients were receiving glatiramer acetate (Copaxone) and 15 were on no immunomodulatory therapy. Unless otherwise indicated, no established relapsing-remitting multiple sclerosis patients were receiving interferon-β1b (Betserson). RA and SLE samples were collected from three different sites in the USA (TN, TX, PA). Relevant institutional board approval from all participating sites was obtained. All subjects provided written informed consent. Additional patient characteristics are summarized in Additional file 1: Table S1.

### siRNA knockdown and cell culture

Cells were cultured in RPMI 1640 medium supplemented with fetal bovine serum (FBS) at 10% (Jurkat) or 20% (THP-1), 1% penicillin/streptomycin, and 1% L-glutamine at 37°C in a humidified atmosphere of 5% CO_2_. Jurkat T cells were obtained from the American Type Culture Collection (ATCC, Manassas, VA). The THP-1 monocytic cell line was from Dr. Jacek Hawiger (Vanderbilt University Medical Center). Single, inventoried silencer select siRNAs (Ambion) against each mRNA were transfected into Jurkat T cells using the Amaxa Cell Line Nucleofector Kit V (Lonza) or into THP-1 cells by Lipofectamine RNAiMAX (Invitrogen).

### RNA isolation, cDNA synthesis, and real-time PCR

Peripheral whole blood was drawn into PreAnalytiX PaxGene tubes (VWR, West Chester, PA, USA). Cell cultures were treated with Tri-Reagent (Molecular Research Center). RNA was isolated following the supplied protocol and purified with the RNEasy MinElute Cleanup kit (Qiagen) and quantified using a Nano Drop 1000 spectrophotometer [46,47]. Complementary DNA (cDNA) was reverse transcribed from total RNA using the SuperScript III First-Strand Synthesis Kit (Life Technologies) using oligo-dT or random hexamer primers and purified using the Qiagen QiaQuick PCR purification kit. Real-time qPCR (Bio-Rad iCycleriQ Real Time PCR System) was performed in duplicate using SYBR green in 15 μL reaction volumes. Primers used in this study are described in Additional file 3: Table S2.

### Western blotting

Western blotting was performed as described previously [48,49]. Briefly, peripheral blood mononuclear cells were isolated from whole blood using BD Vacutainer Cell Preparation Tubes. Whole cell lystates were resolved by SDS polyacrylamide gel electrophoresis and transferred to polyvinylidene fluoride (PVDF) membranes overnight at 4°C. Membranes were washed and blocked using Odyssey Blocking Buffer (Li-COR Biosciences, Lincoln, NE, USA) for 1 h at room temperature. Membranes were rinsed and incubated with primary antibodies overnight at 4°C. Antibodies used in this study: monoclonal mouse anti-SSB (ab75927; Abcam), polyclonal rabbit anti-TROVE2 (NBP1-86998; Novus Biologicals), and mouse monocloncal anti-actin (sc-8432; Santa Cruz Biotechnology). Membranes were then washed and incubated with fluorescently labled IRDye 700/800 antibodies diluted in blocking buffer in the dark. Blots were washed and resuspended in TBS prior to scanning and band quantification using the Li-COR Odyssey Infrared Imaging System (Li-COR Biosciences, Lincoln, NE, USA).

### RNA-Immunoprecipitation (RIP)

RIP analysis was performed as described previously [50,51]. Briefly, Jurkat T cells were harvested, nuclei isolated, lysed, and chromatin sheared, followed by incubation with monoclonal mouse anti-SSB (ab 75927; Abcam) or polyclonal rabbit anti-TROVE2 (NBP1-86998; Novus) overnight at 4°C. Protein A/G beads were added to the lysate and incubated at 4°C for an additional 3 h. Beads were pelleted, supernatants harvested, and beads washed and suspended in Tri-Reagent. RNA bound to the immunoprecipitate was purified following the manufacturer’s supplied protocol (Molecular Research Center).

### RNA-seq sample preparation and data analysis

We extracted RNA from healthy controls (N = 8) and established relapsing-remitting multiple sclerosis patients (N = 6) using PaxGene tubes according to the manufacturer’s protocol. Library preparation was then performed using the Illumina Tru-Seq RNA kit using oligo-dT primers. RNA-sequencing was conducted in the Vanderbilt Technologies for Advanced Genomics (VANTAGE) core. One hundred bp paired-end reads were generated with an Illumina HiSeq 2500. A quality control step was initially performed on the raw data to identify potential outliers before any advanced analysis using tools such as Fastx Toolkit and FastQC [52]. The RNA data were aligned with TopHat and gene expression levels were quantified using Cufflinks [53,54]. FPKM (fragments per kilobase per million reads) based approaches (Cuffdiff) were used to detect differentially expressed genes [55]. False discovery rate (FDR <0.05) was used for multiple test correction.

MatLab (version R2013a) was used to construct a matrix of counts from the data. In particular, MatLab functions included in the *Bioinformatics Toolbo*x (for example, getCounts, estimateBaseParams, computePVal, and so on) were employed to extract counts on basepair (bp) intervals specified by library GTF annotation files or by customized un-annotated locations along each chromosome in annotated exons and introns. These raw counts were normalized using the medians of the sets formed by the ratios of the raw counts and the non-zero geometric mean of the raw counts on each interval. These normalized counts were then used to perform the statistical analysis comparing the control and multiple sclerosis groups. In particular, the *P* values and adjusted *P* values for the difference of the means of the two groups were computed on each bp interval. The Benjamini-Hochberg procedure was used to find the adjusted *P* value and correct for FDRs from multiple sampling. Next, the log_2_ of the ratio of the mean normalized multiple sclerosis counts to the mean normalize control counts was computed for bp intervals that had non-zero geometric means. Lastly, bp intervals where the adjusted *P* value was less than a prescribed number (for example, 0.01) and the absolute value of the log_2_ ratio was larger than a second prescribed number (for example, 4) were then selected for further analysis. Output from MatLab calculations were inputted into *Mathematica* (version 9.0.1) whose GeonomeData database was used to further explore coding and non-coding regions along the chromosomes. We considered loss of one or more exons at the 5’ end of an individual mRNA transcript to be an example of 5’ mRNA shortening and loss of one or more exons at the 3’ end of an individual mRNA to be an example of 3’ mRNA shortening. Intron retention was defined as gain of specific RNA transcripts within introns.

### Statistical analysis

Data are expressed as the mean ± SD of three or more independent experiments. Significance was determined by Student’s *t*-test using GraphPad Prism Software (La Jolla, CA, USA). *P* values <0.05 were considered significant.

### Data availability

The RNA-sequencing data used in this study are accessible through NCBI’s Gene Expression Omnibus using accession code GSE66573.

## Additional files

Additional file 1: Table S1. Summary of patient characteristics. Additional file 2: Figure S1. RNA-immunoprecipitation analysis of target mRNA binding to Ro60 and La proteins. Ro60 or La proteins were immunoprecipitated with specific antibodies. Levels of each mRNA (X-axis) were determined by quantitative PCR using exon-specfic primers described in Figure 4F. Result are expressed as fraction of the total of each mRNA unbound or recovered in the Ro60 or La immunoprecipitates (± S.D). Additional file 3: Table S2. Primers used in quantitative PCR reactions.

## Abbreviations

CIS: Clinically isolated syndrome
ncRNA: Non-coding RNA
NMO: Neuromyelitis optica
PD: Parkinson’s disease
RA: Rheumatoid arthritis
RRMS: Relapsing remitting multiple sclerosis
SLE: Systemic lupus erythematosus
