# Supplementary material for: Defective structural RNA processing in relapsing-remitting multiple sclerosis
Source: Genome Biol. 2015 Mar 25;16(1):58. doi: 10.1186/s13059-015-0629-x (PMC4403723; doi:10.1186/s13059-015-0629-x)
Supplement: Additional file 1: Table S1. — Summary of patient characteristics. [file 13059_2015_629_MOESM1_ESM.docx]

**Additional file 1: Table S1. Summary of Patient Characteristics**

**(A)**

Controls MS-CIS MS-NAIVE RRMS NMO RA SLE PD

(n=96) (n=26) (n=34) (n=94) (n=22) (n=18) (n=24) (n=19)

Age, mean ± SD yrs. 38 ± 11 38 ± 10 46 ± 10 43 ± 9 45 ± 9 51 ± 14 42 ± 13 60 ± 14

Female (%) 73 88 84 82 80 94 94 84

Ethnicity (%)

Caucasian 58 81 80 76 64 83 45 84

African American 22 15 20 24 36 11 33 16

Hispanic 13 4 -- -- -- 6 11 --

Asian 7 -- -- -- -- -- 11 --

Disease Duration, -- 0.7 ± 0.6 -- 7 ± 5 5 ± 4 11 ± 9 6 ± 4 4 ± 3

(mean years ± SD)

*Except where indicated otherwise, values are the percent.

**(B)**

**Western Blot** Controls RRMS

(n=9) (n=8)

Age, mean ± SD yrs. 39 ± 17 42 ± 10

Female (#) 7 8

Ethnicity (#)

Caucasian 7 7

African American 2 1

Hispanic -- --

Asian -- --

Disease Duration, -- 12 ± 9

(mean years ± SD)

**(C)**

**RNA-sequencing** Controls RRMS

(n=8) (n=6)

Age, mean ± SD yrs. 34 ± 6 34 ± 8

Female (#) 6 4

Ethnicity (#)

Caucasian 8 6

African American -- --

Hispanic -- --

Asian -- --

Disease Duration, -- 3 ± 1

(mean years ± SD)
