# Supplementary material for: Defective structural RNA processing in relapsing-remitting multiple sclerosis
Source: Genome Biol. 2015 Mar 25;16(1):58. doi: 10.1186/s13059-015-0629-x (PMC4403723; doi:10.1186/s13059-015-0629-x)
Supplement: Additional file 3: Table S2. — Primers used in quantitative PCR reactions. [file 13059_2015_629_MOESM3_ESM.docx]

**Additional file 3: Table S2: Primers used in quantitative PCR reactions.**

**GAPDH For** AGCCACATCGCTCAGACAC

**Rev** GCCCAATACGACCAAATCC

**RNY1 For** GGCTGGTCCGAAGGTAGTGA

**Rev** GCAGTAGTGAGAAGGGGGGA

**RNY3 For** GGCTGGTCCGAGTGCAGTGG

**Rev** GAAGCAGTGGGAGTGGAGAA

**RNY4 For** GGCTGGTCCGATGGTAGTGG

**Rev** TTAGCAGTGGGGGGTTGTAT

**RNY5 For** AGTTGGTCCGAGTGTTGTGG

**Rev** AACAGCAAGCTAGTCAAGCG

**TROVE2 For** tcacatcttaaaccttccagtga

**Rev** gagagtgctttttctttatacaattca

**SSB For** gatgaaaatggtgcaactgg

**Rev** ctgttttctgttgtttggatgc

**U1 For** tttttccagagcgaggctta

**Rev** ccacaaattacgcagtcgag

**U1 1 For** aaaaagggcttctgtcgtga

**Rev** atcaccagctgcccaaatac

**U12 For** aaaataacgattcggggtga

**Rev** caggcatcccgcaaagtag

**CSF1R-Exon 2** **For** ccagtgcagaggagaggaac

**Rev** cagcaggagcagcagaactc

**CSF1R-EXON 22** **For** tgcagcccaacaactatcag

**Rev** cctcaccttcccaagtttca

**MBP-EXON 1 For** tgggtgcgcgcccgtccctcggagccgccg

**Rev** cggcgctgcgctcgttgctccgaggccgag

**MBP-EXON 4** **For** gagaggcagatgcgaaccagaacaatggga

**Rev** cttgccagagccccgcttgggcgcacccct

**MBP-INTRON** **For** gctgtctcctgtgcctggcccggtgtccat

**Rev** caagaacttggggtcccttcccagagccag

**NFATC1-EXON 2** **For** gtgccacaaccttcagacct

**Rev** gtcttccacctccacatcgt

**NFATC1-EXON 9** **For** ccaggggttaagtcctctcc

**Rev** gagaaaggtcgtggagcttg

**NFATC1-SHORT For** gtcctgtctggccacaactt

**Rev** gggatctcaaccaccagaga

**NFATC1-LONG For** gtcctgtctggccacaactt

**Rev** gggagtccgaggtgacagt

**18S rRNA 28s rRNA**

**5’-30** TCGGGGCCTCGCCGCGCTCT TCCGTCCGCCGAGCGGCCCGT

**5’-60** CCGCGGCCCGTCCGTCCGTC TCTCTCTCCCGTCGCCTCT

**5’-90** CGCCCCGGCCCCACCGGT CCTCCGGTCGTCCCGCT

**5’-0** GCCATGCATGTCTGAGTACG GACCCGCTGAATTTAAGCAT

**5’-100** TCTGATAAATGCACGCATCC GCCTCGATCAGAAGGACTTG

**3’-0** GATCCTTCCGCAGGTTCAC CTTGTGTCGAGGGCTGACTT

**3’-100** CGCTACTACCGATTGGATGG GGCGCTAAACCATTCGTAGA

**3’+30** GCCGCGGGCCTCGCCCT CCCCGCCGGGCCCCCCGCA

**3’+60** TGGGTGTGCGGAGGGAA CGGGAGGGAGGAAGACGA

**3’+90** GAGCGAACGAACGGGCA ACCGGGGTCGCGCGCCCT
